# Design Optimization and Fabrication of High-Sensitivity SOI Pressure Sensors with High Signal-to-Noise Ratios Based on Silicon Nanowire Piezoresistors

**DOI:** 10.3390/mi7100187

**Published:** 2016-10-14

**Authors:** Jiahong Zhang, Yang Zhao, Yixian Ge, Min Li, Lijuan Yang, Xiaoli Mao

**Affiliations:** 1 Jiangsu Key Laboratory of Meteorological Observation and information Processing, Nanjing University of Information Science and Technology, Nanjing 210044, China; geyixian@nuist.edu.cn (Y.G.); maoxiaoli@nuist.edu.cn (X.M.); 2 Jiangsu Collaborative Innovation Center on Atmospheric Environment and Equipment Technology, Nanjing University of Information Science and Technology, Nanjing 210044, China; 3 School of Electronic and Information Engineering, Nanjing University of Information Science and Technology, Nanjing 210044, China; zhaoyang@nuist.edu.cn; 4 School of Information Science and Technology, Suqian College, Suqian 223800, China; ljyang@sqc.edu.cn

**Keywords:** silicon nanowire, piezoresistive pressure sensor, high-sensitivity, high signal-to-noise ratio, optimized design, fabrication process, data fusion, particle swarm optimization–back-propagation (PSO–BP) neural network, temperature drift compensation

## Abstract

In order to meet the requirement of high sensitivity and signal-to-noise ratios (SNR), this study develops and optimizes a piezoresistive pressure sensor by using double silicon nanowire (SiNW) as the piezoresistive sensing element. First of all, ANSYS finite element method and voltage noise models are adopted to optimize the sensor size and the sensor output (such as sensitivity, voltage noise and SNR). As a result, the sensor of the released double SiNW has 1.2 times more sensitivity than that of single SiNW sensor, which is consistent with the experimental result. Our result also displays that both the sensitivity and SNR are closely related to the geometry parameters of SiNW and its doping concentration. To achieve high performance, a p-type implantation of 5 × 10^1^^8^ cm^−3^ and geometry of 10 µm long SiNW piezoresistor of 1400 nm × 100 nm cross area and 6 µm thick diaphragm of 200 µm × 200 µm are required. Then, the proposed SiNW pressure sensor is fabricated by using the standard complementary metal-oxide-semiconductor (CMOS) lithography process as well as wet-etch release process. This SiNW pressure sensor produces a change in the voltage output when the external pressure is applied. The involved experimental results show that the pressure sensor has a high sensitivity of 495 mV/V·MPa in the range of 0–100 kPa. Nevertheless, the performance of the pressure sensor is influenced by the temperature drift. Finally, for the sake of obtaining accurate and complete information over wide temperature and pressure ranges, the data fusion technique is proposed based on the back-propagation (BP) neural network, which is improved by the particle swarm optimization (PSO) algorithm. The particle swarm optimization–back-propagation (PSO–BP) model is implemented in hardware using a 32-bit STMicroelectronics (STM32) microcontroller. The results of calibration and test experiments clearly prove that the PSO–BP neural network can be effectively applied to minimize sensor errors derived from temperature drift.

## 1. Introduction

To date, the microelectromechanical system (MEMS) silicon piezoresistive pressure sensors have been used in a diverse range of commercial and engineering applications including consumer, automobiles, biomedicine, process control, military, meteorology, and aerospace industry areas [1,2,3,4,5,6]. This is mainly attributed to the fact that they have significant advantages, including low cost, low energy consumption, wide dynamic range, mass producibility, etc. However, the piezoresistive pressure sensors have disadvantage over their large size, low sensitivity and poor signal-to-noise ratios in contrast with high performance of the micromachined capacitive and resonant pressure sensors [5,6,7]. To overcome these major problems, many design principles and considerations as well as optimization methods have also been proposed [8,9,10], yet they basically have some inevitable drawbacks as summarized in the available published literature [5,6].

With the rapid development of micro/nano processing technology, recently, there have been studies trying to reduce the sizes of conventional MEMS piezoresistive pressure sensors and enhance their low sensitivity by using a wide variety of nanomaterials and nanostructures as sensing elements [7,11,12,13,14,15,16], such as silicon nanorods and silicon nanowires (SiNWs), which have the small size and ultra-high piezoresistive effect [17,18,19,20,21,22,23,24]. For examples, Kim et al. [7] and Soon et al. [11] reported the high-sensitivity piezoresistive pressure sensors using the high piezoresistive effect of the SiNWs, respectively. Lou et al. [12] proposed a nanoelectromechanical system (NEMS)-based piezoresistive pressure sensor by utilizing the SiNWs as the sensing elements, which has relatively high sensitivity of about 0.6% psi^−1^. Nevertheless, it is noteworthy that tradeoffs should be made in the optimization design of NEMS piezoresistive pressure sensors. Obviously, noise determines the minimum detection signal, which is a very important factor in the performance of the pressure sensors, so the presence of noise is the limiting factor in the sensor design and also needs to be carefully considered [5,6,25]. In other words, the sensor design parameters must be properly chosen to balance the pressure sensitivity and voltage noise sources of the NEMS piezoresistive pressure sensor given a set of design and operating constraints, especially where a high signal-to-noise ratio (SNR) is required for the faithful measurement of the small pressure differentials [26]. However, an exhaustive analysis considering the influences of doping concentration and the geometry of SiNW piezoresistors on optimizing the performance of the NEMS piezoresistive pressure sensors in terms of sensitivity and signal-to-noise ratio has been rarely reported till now.

In this paper, in order to obtain high-performance piezoresistive pressure sensors based on silicon on insulator (SOI), we investigate the design, optimization modeling, fabrication, measurement and temperature drift compensation of the NEMS pressure sensor taking into account the balance between the low voltage noise and the high pressure sensitivity. Specifically, the double single-crystal SiNW piezoresistors with the optimal size and doping concentration are introduced as the sensing structures for our piezoresistive pressure sensors made of SOI wafer. The SiNWs not only decrease the size and enhance the sensitivity because of their high piezoresistive effect but also balance the voltage noise and the sensitivity. The different noise components commonly present with the piezoresistive-type sensors are carefully considered and modeled so as to improve the SNR. It is noteworthy that using SOI material can reduce the leakage current through the dielectric isolation instead of P-N isolation and increase the operation temperature of the pressure sensor. Meanwhile, the fabrication process for the SOI-based piezoresistive pressure sensor is also compatible with complementary metal-oxide-semiconductor (CMOS)-MEMS technology. However, the performance of piezoresistive pressure sensor is highly dependent on temperature variation primarily due to the change in the piezoresistive coefficient with temperature. Temperature affects the piezoresistive coefficient through a change in the mobility and carrier concentration in the respective bands [5,24]. Unfortunately, the temperature drift of sensitivity is more serious for silicon nanostructures [27]. For the sake of eliminating the effects of temperature and improve measurement accuracy, the back-propagation (BP) neural network improved by the particle swarm optimization (PSO) algorithm [28,29] is applied to achieve the temperature drift compensation in the study.

## 2. Configuration of the SiNW Pressure Sensor and Basic Theory

### 2.1. Structure of the SiNW Pressure Sensor

**Figure 1:**
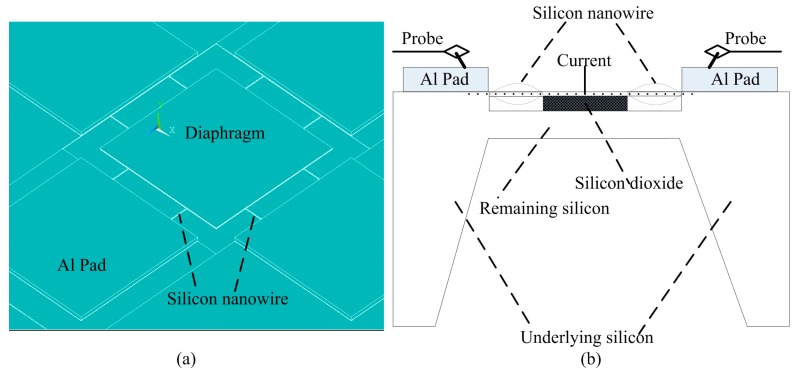
(**a**) Schematic diagram of the nanoelectromechanical system (NEMS) piezoresistive pressure sensor using double silicon nanowire (SiNW) piezoresistors; and (**b**) the measurement principle of the SiNW pressure sensor.

According to previous literature [17,18,19,20,21,22,23,24,30], the SiNW under the thickness of 340 nm may exhibit a good or giant piezoresistive effect. For instance, when the size is made about 140 nm × 200 nm, SiNW has seven times more piezoresistive effect than that of bulk silicon [30]. To reduce the sensor size and improve the sensitivity, the piezoresistive pressure sensor based on SOI utilizing double released SiNW with high piezoresistive effect is proposed in this paper. Figure 1a displays the schematic of the proposed SOI piezoresistive pressure sensor using double silicon naowires along <110> direction. The double SiNW piezoresistors (four pairs) are connected like a bridge between the middle silicon diaphragm and the edge of silicon substrate. As we known, the fundamental concept of piezoresistive effect is the change in the electrical resistivity of a semiconductor material resulting from an applied stress and it is commonly employed in pressure sensors. That is to say when pressure applies on the silicon diaphragm (freely suspended membrane) of the sensor, these SiNWs around diaphragm receive stress to change their resistance. The changed resistance perceives adopting the fixed current signal passed through the double nanowires and diaphragm. The specific measurement principle of the piezoresistive pressure sensor proposed by us is shown in Figure 1b. The SiNWs position and the sizes of both SiNWs and the proposed pressure sensor should be determined carefully by using the ANSYS finite element simulation (version 14.5, ANSYS Inc., Canonsburg, PA, USA) so that the high sensitivity can be obtained. Generally, the SiNWs should be located to receive the maximum stress based on the simulation result.

### 2.2. The Sensitivity of the SiNW Pressure Sensor

The sensitivity is a significant indicator of the piezoresistive pressure sensor. It depends on the initial zero-stress resistance *R* of the piezoresistors, which is determined by its size and doping concentration [5]. For a SiNW piezoresistor with area *A_R_*, the piezoresistive pressure sensitivity *S* is given by

(1) $$S=\frac{\Delta R/R}{\Delta P}=\frac{1}{A_{R}\Delta P}\int_{0}^{A_{R}}{(\pi }_{L}\sigma_{L}+\pi_{T}\sigma_{T})\partial A$$

where Δ*R* represents the resistance change of the piezoresistor under the differential stress Δ*P*. π*_L_* and π*_T_* refer to the longitudinal piezoresistive coefficient (for stress applied parallel to the current flow in the piezoresistor) and transverse piezoresistive coefficient (for stress applied perpendicular to the current flow), respectively, while σ*_L_* and σ*_T_* are the longitudinal and transverse stress in the SiNW piezoresistors formed by the external pressure Δ*P*. For the NEMS piezoresistive pressure sensor with complex structure, the stress *σ* of its sensitive element is obtained by ANSYS finite element simulation.

According to Equation (1), for the purpose of investigating the relationship between the sensitivity and doping concentration as well as temperature, the dependence of the piezoresistive coefficient (π) as functions of the doping concentration (*n*) and temperature (*T*) is taken into account. The piezoresistive coefficient π(*n*,*T*) of the SiNW piezoresistor obeys to the following relation [5]:

(2) $$\pi (n,T)=\pi (n_{0},T_{0})\frac{300}{T}\frac{F_{-\frac{3}{2}}(E_{F}/K_{B}T)}{F_{-\frac{1}{2}}(E_{F}/K_{B}T)}$$

The coefficient π(*n_0_*,*T_0_*) stands for the piezocoefficient value for the SiNW of low doping concentration (*n_0_*) at room temperature (*T_0_*). The Fermi integral is the function of temperature (*T*) and the Fermi energy (*E_F_*), and *K_B_* denotes the Boltzmann constant. The Fermi energy is determined from *n*. It is found that the piezoresistive effect significantly decreases at high temperature and doping concentration due to carrier-phonon scattering as well as carrier-carrier scattering. In fact, the piezoresistive coefficients of the SiNWs are sensitive to many other quantities such as size, orientation and band structure [31,32,33,34]. According to the published papers [17,18,19,20,21], the SiNWs have giant piezoresistive effect. The reason for higher piezoresistance is thanks to reduced dimensions, increased surface depletion region and enhanced surface trapping effect under pressure [17,20,23,24,35].

### 2.3. The SNR of the SiNW Pressure Sensor

Noise is one of the most important factors limiting the sensitivity of piezoresistive pressure sensor. The noise power spectral density of the nanowire piezoresistor $V_{\mathrm{noise}}^{2}$ is mainly composed of Johnson noise power spectral density $V_{J}^{2}$ and flicker (1/*f*) noise power spectral density $V_{f}^{2}$ [5,6,36,37], which can be described as:

(3) $$V_{\mathrm{noise}}^{2}=V_{J}^{2}+V_{f}^{2}$$

Johnson noise generates in resistors owing to random motion (thermal agitation) of carriers and is independent of frequency. In the pressure sensor using double SiNW, Johnson noise is closely related to the resistance and temperature of the nanowire, which can be expressed as [5,6,36,37]:

(4) $$V_{J}=\sqrt{4K_{B}TR}=\sqrt{\frac{4K_{B}T(l+w)}{nq\mu wt}}$$

where *K_B_* denotes the Boltzmann constant, *T* is the temperature, *R* is the resistance of the double SiNW piezoresistor, *n* is the carrier’s concentration, *q* is the electron charge, μ is the hole mobility and *l*, *w*, and *t* are the length, width, and thickness of the nanowire piezoresistor, respectively.

The dominant 1/*f* noise source in silicon piezoresistors is Hooge noise [9,36], which is a fluctuation in resistor conductance caused by drawbacks in the bulk of the material. Different from Johnson noise, it is a conductivity voltage noise that depends on the bias voltage. For the case of applied current, the 1/*f* noise is given by the following equation:

(5) $$V_{f}=\sqrt{\frac{\alpha }{Nf}}IR=\sqrt{\frac{\alpha }{(4nlwt+tb^{2})\;f}}\frac{I(l+w)}{nq\mu wt}$$

where *I* is the bias current applied to the nanowire with the total number of carriers *N*, *f* is the frequency, *b* is the size of suspended membrane, α is the Hooge factor, which is a device dimension-independent parameter, and is between 3.2 × 10^−6^ and 5.7 × 10^−6^ in single crystal silicon [38].

SNR is a key indicator of the pressure sensor and for the case when the constant current is applied, and it may ultimately be written for the quarter Wheatstone bridge circuit as:

(6) $$\mathrm{SNR}=20\log\frac{V_{\mathrm{out}}}{V_{\mathrm{noise}}}=20\log\frac{I(\pi_{L}\sigma_{L}+\pi_{T}\sigma_{T})R}{4\sqrt{V_{J}^{2}+V_{f}^{2}}}=20\log\frac{I(\pi_{L}\sigma_{L}+\pi_{T}\sigma_{T})}{\sqrt{\frac{64K_{B}Tnq\mu wt}{l+w}+\frac{16\alpha I^{2}}{(4nlwt+tb^{2})\;f}}}$$

## 3. Sensor Design

The target of design and optimization for the piezoresistive pressure sensor is to maximize its performance, that is to say, both the signal-to-noise ratio and sensitivity of the sensor should meet the desired specifications. In the following sections, we present an analysis of the sensor model design, pressure sensitivity, voltage noise and the signal-to-noise ratio.

### 3.1. Design Optimization Based on Finite Element Simulation

**Figure 2:**
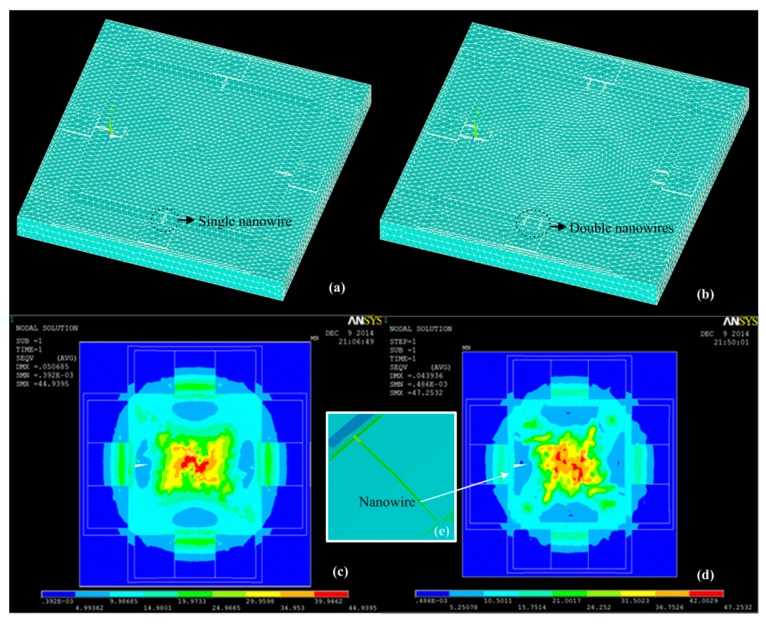
(**a**) Three-dimensional finite element mesh of single SiNW piezoresistive pressure sensor; (**b**) three-dimensional finite element mesh of double SiNW piezoresistive pressure sensor; (**c**) average stress distribution of single SiNW pressure sensor; (**d**) average stress distribution of double SiNW pressure sensor; and (**e**) average stress distribution of the SiNW.

The expected working range of the designed sensor using SiNWs is 0–0.1 MPa. To decide the specific optimized structures, ANSYS finite element simulations are firstly performed. Three-dimensional finite element mesh and the average stress distribution of single and double SiNW pressure sensors by using ANSYS simulation under 0.1 MPa are plotted in the Figure 2. From the simulation results, it is found that the double SiNW exhibits greater stress and the stress of the nanowire is substantially uniform, thus avoiding the significant nonlinear error.

**Figure 3:**
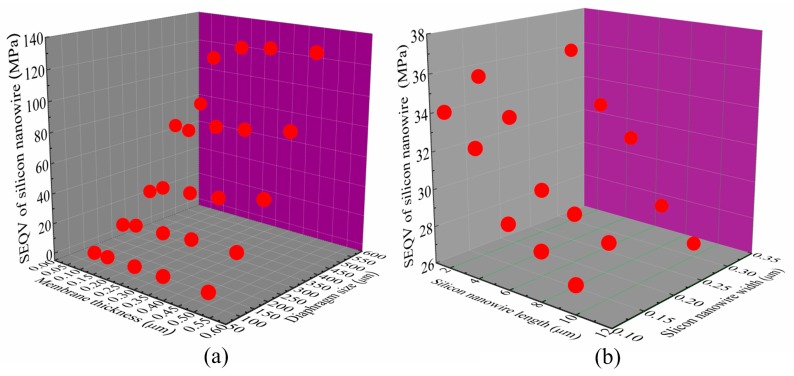
ANSYS simulation results: (**a**) the von Mises equivalent stress (SEQV) of double SiNW of 2 µm length vs. diaphragm size and membrane thickness; and (**b**) SEQV of double SiNW vs. the length and width of double SiNW.

Figure 3 presents the von Mises equivalent stress (SEQV) of SiNW along the <110> direction as a function of structural parameters of the SiNW pressure sensor under 0.1 MPa applied stress, such as the diaphragm size, the thickness of membrane top-layer, and the length and width of the nanowire. As we can see in Figure 3a, the effect of the thickness of membrane top-layer on the SEQV is relatively small. However, the SEQV of double SiNW rapidly and monotonically increases with the increase of the diaphragm size. The fracture stress of silicon is about 300 MPa. For practical purposes, it is advisable to consider the maximum stress that can be developed within the chip as one fifth of the fracture stress [5], thus the proposed piezoresistive pressure sensor is designed to have the diaphragm size of less than 300 µm × 300 µm [7,12]. In addition to the diaphragm size, the size of the SiNW is also important parameter for sensor design. From the ANSYS simulation results plotted in Figure 3b, it is found that the influence of the width of the nanowire on the SEQV is small and the trend is monotonic with the length of the SiNW. Besides, it is concluded that one obtains the relatively optimal SEQV value when the length of SiNW varies from 2 to 10 µm, which is consistent with the result reported in the literature [12].

**Figure 4:**
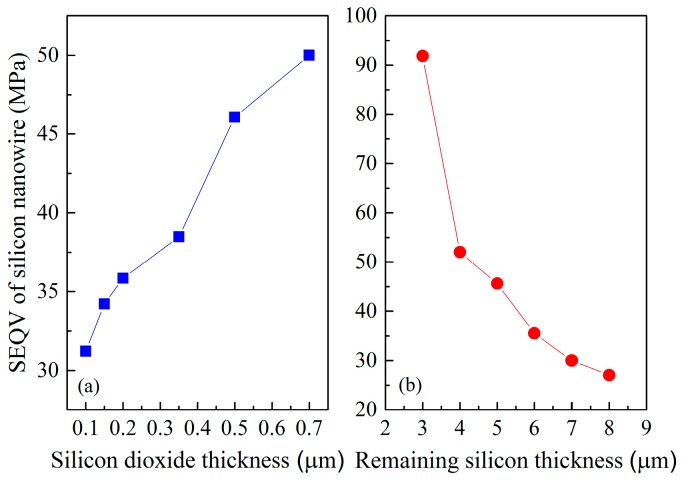
ANSYS simulation results: (**a**) SEQV of SiNW vs. silicon dioxide thickness; and (**b**) SEQV of SiNW vs. the remaining underlying silicon thickness.

After determining the diaphragm size and SiNW dimension, the next step is to explore the relationships of the SEQV of SiNW with respect to silicon dioxide thickness and the thickness of the remaining silicon as a result of backside of silicon substrate was etched, as shown in Figure 1b. Figure 4a indicates that the thicker silicon dioxide layer is, the larger SEQV is. Figure 4b illustrates that the SEQV rapidly increases with the decrease of the thickness of the remaining silicon, but the thickness should be greater than 4 µm. However, to obtain high maximum stress, one should also take production issues into account. Considering the stress simulation, safety limitations of fabrication process and published high piezoresistive effect of nanowire, the proposed high performance piezoresistive pressure sensor is designed to have the nanowire size under 1400 nm × 100 nm, the nanowire length of 10 µm, the membrane top-layer thickness of 100 nm, the middle silicon dioxide layer thickness of 200 nm, the diaphragm size of 200 µm × 200 µm, and the remaining silicon thickness of 6 µm. Of course, we need to combine the performance of the sensor such as sensitivity and SNR to further analyze whether these geometric sizes of the sensor are optimal. The work is completed in the next section.

### 3.2. Sensor Sensitivity and SNR Analysis

**Figure 5:**
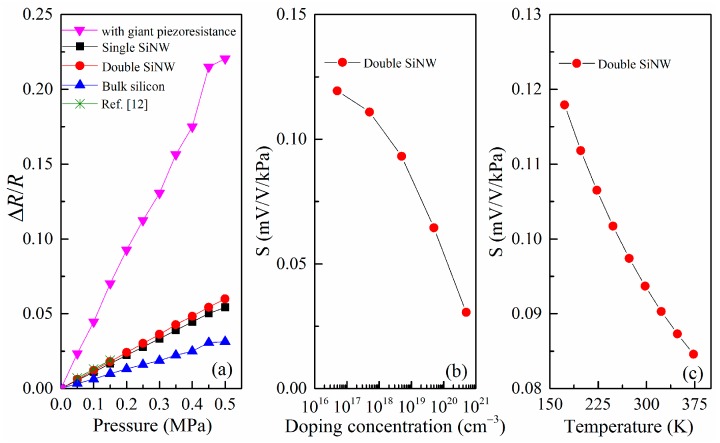
(**a**) Relative resistance change as a function of the applied pressure for single and double SiNW and bulk silicon; (**b**) variation of the sensitivity of double SiNW pressure sensor with the doping concentration; and (**c**) variation of the sensitivity of double SiNW pressure sensor with the temperature.

To understand the enhanced sensitivity, we compare the designed SOI pressure sensor using double SiNW with pressure sensors using single SiNW and bulk silicon. The simulation results obtained from ANSYS stress analysis and Equation (1) at room temperature are shown in Figure 5a, which demonstrates the change in resistance with the applied pressure in the range of 0 to 0.5 MPa. It is observed that resistance change of the pressure sensor of the released double SiNW is consistent with that of a similarly-sized SiNW pressure sensor reported by Lou, et al. [12]. As a result, sensor of the released double SiNW with the proposed sizes has sensitivity of 120 mV/V·MPa and it has 1.2 times more sensitivity than one of the pressure sensor of the released single SiNW and has two times more sensitivity than bulk silicon sensor that is not released. It is worth noting that the increase in sensitivity is mainly due to the released SiNW with small sizes provides a stress concentration enhanced structure [39]. If considering the high piezoresistive characteristics of SiNW previously prepared by us [21], the sensitivity will further significantly increase to about 450 mV/V·MPa.

Based on microscopic piezoresistive model we previously proposed [32], the relationship between sensitivity and doping concentration of SiNW is also calculated using Equations (1) and (2). According to Figure 5b, the sensitivity decreases monotonically with the doping concentration, which is mainly owing to the piezoresistive coefficient decreases with increasing doping concentration. Therefore, for the design of high-sensitivity miniature pressure sensor, the doping concentration should not be too high. Meanwhile, the doping concentration cannot be too low. On the one hand, it is difficult to form an Ohmic contact when the doping concentration is low. On the other hand, the sensor noise and temperature drift increase with the doping concentration decreasing, thus affecting the overall SNR. Therefore, the selection of the doping concentration should be comprehensively considered.

In addition, the sensitivity of piezoresistive pressure sensors based on SiNWs has a thermal drift because the temperature obviously affects the piezoresistive coefficient and the individual values of resistances of the Wheatstone bridge [9,27]. Thus, the temperature coefficient of sensitivity (TCS) depends on both the temperature coefficient of piezoresistance (TCπ) and the temperature coefficient of resistance (TCR). In the case, the sensor should be temperature compensated both from the effect of change in the resistance value and that in the piezoresistive coefficient. Taking into account that TCπ is a negative quantity but TCR is a positive quantity, it is possible to choose an appropriate doping concentration at which both of them can theoretically cancel each other, causing an internal temperature compensation of the sensor [9]. In this paper, the TCR component of sensitivity change is mainly compensated by using a Wheatstone bridge circuit with a negative-temperature-coefficient resistance, while the TCπ component is compensated by using the PSO–BP data fusion algorithm. Figure 5c gives the theoretical calculation results for the relationship between the sensitivity and the temperature. The sensitivity decreases monotonically with the temperature increases, which is mainly attributed to the piezoresistive coefficient of the SiNW piezoresistor decreases with increasing the temperature [27]. A clear nonlinear relationship is observed as the temperature increases, and the TCS extracted is around −1.7 × 10^−4^ K^−1^ by linear fitting.

**Figure 6:**
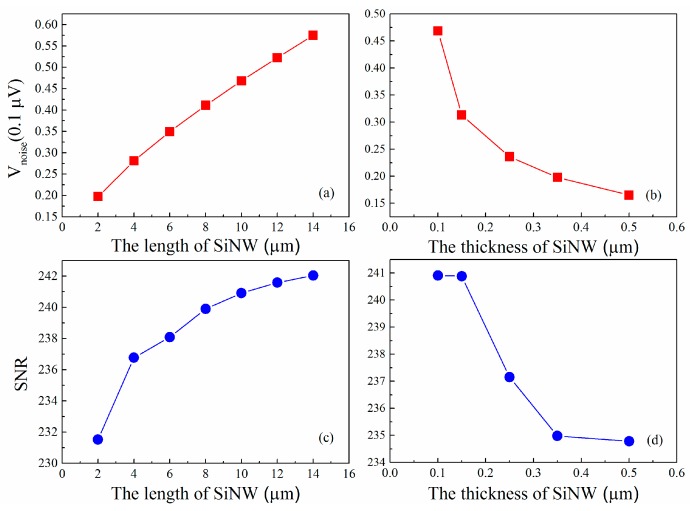
(**a**) Variation of the voltage noise with the length of SiNW; (**b**) variation of the voltage noise with the thickness of SiNW; (**c**) variation of SNR with the length of SiNW; and (**d**) variation of SNR with the thickness of SiNW.

**Figure 7:**
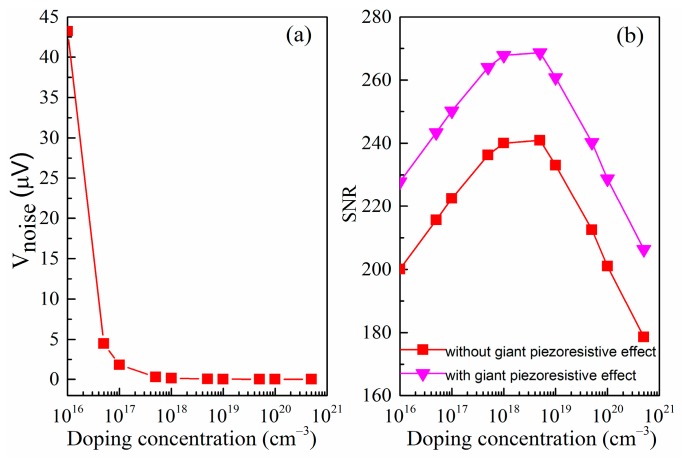
(**a**) Variation of the voltage noise with doping concentration of the SiNW piezoresistor; and (**b**) variation of SNR with doping concentration of the SiNW piezoresistor.

In the following section, the influencing factors of noise and SNR of nanowire pressure sensor are analyzed by using the above noise models (Equations (3)–(6)). The variation of the voltage noise and SNR with the size of SiNW piezoresistor and the doping concentration are plotted in Figure 6 and Figure 7, respectively. For the constant-current case, Figure 6a,b reveals that the noise voltage of the double SiNW is proportional to the length of SiNW as well as inversely proportional to the thickness of SiNW. It is interesting that SNR follows a similar law. A long and thin SiNW has the better signal-to-noise ratio, which is consistent with the results of the literature [6,38]. In Figure 6c,d, it is also observed that the proposed SiNW with 10 µm length and 100 nm thickness exhibits an excellent signal-to-noise ratio.

As can be seen in Figure 7a, the noise voltage is inversely proportional to the doping concentration and it changes very severely at low concentrations but it changes very slowly at high concentrations. In addition, it can be found that the SNR firstly increases with doping concentration increasing because the flicker noise voltage varies inversely. However, the SNR subsequently decreases since the Johnson noise voltage tends to dominate and piezoresistive coefficient is also reduced. It is apparently noted that the noise minimum and signal-to-noise ratio maximum in the doping concentration range of 1 × 10^1^^8^ to 5 × 10^1^^8^ cm^−3^. Taking into account the good Ohmic contact and the low power consumption of SiNW pressure sensor, p-type implantation of 5 × 10^1^^8^ cm^−3^ is then chosen in this paper. In Figure 7b, we can also find that the SNR has been improved after considering the giant piezoresistive effect. In short, our work shows that we can optimize the design to reduce the noise and improve the signal-to-noise ratio of piezoresistive pressure sensor using SiNW. We hope that the results of these theories can be helpful for future design of experiment methods to achieve the most optimized sensor.

## 4. Fabrication Process of SOI Piezoresistive Sensor

**Figure 8:**
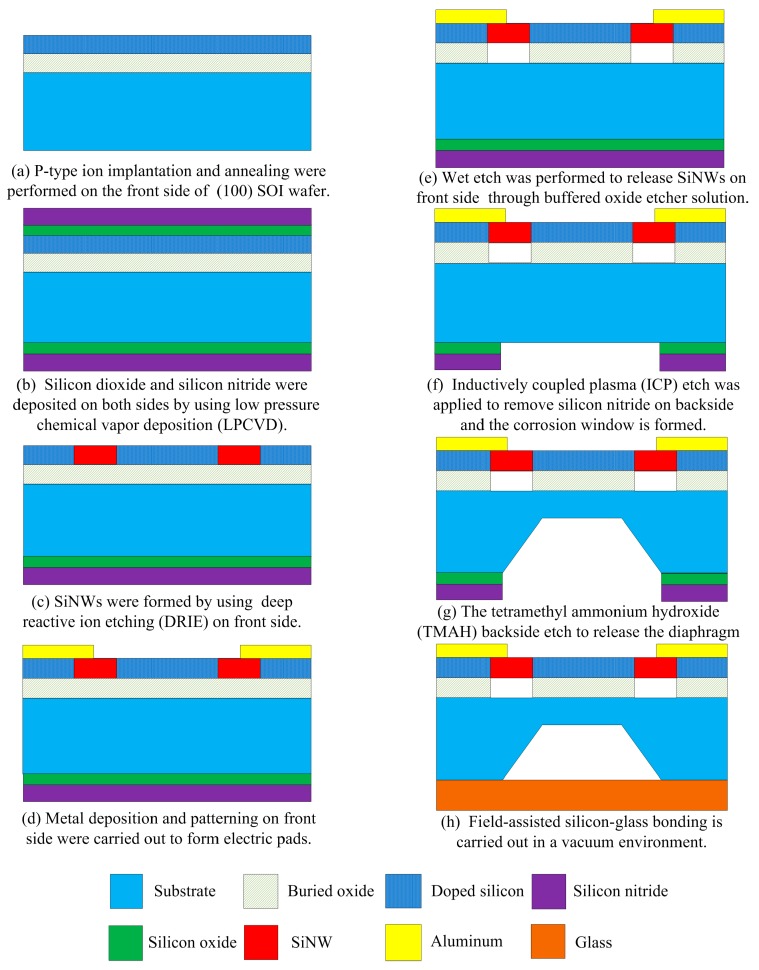
Process flow of the proposed SiNW piezoresistive pressure sensor (the drawing is not to scale). (**a**) P-type ion implantation and annealing were performed on the front side of (100) silicon on insulator (SOI) wafer; (**b**) Silicon dioxide and silicon nitride were deposited on both sides by using low pressure chemical vapor deposition (LPCVD); (**c**) SiNWs were formed by using deep reactive ion etching (DRIE) on front side; (**d**) Metal deposition and patterning on front side were carried out to form electric pads; (**e**) Wet etch was performed to release SiNWs on front side through buffered oxide etcher solution; (**f**) Inductively coupled plasma (ICP) etch was applied to remove silicon nitride on backside and the corrosion window is formed; (**g**) The tetramethyl ammonium hydroxide (TMAH) backside etch to release the diaphragm; (**h**) Field-assisted silicon-glass bonding is carried out in a vacuum environment.

The proposed SiNW piezoresistive pressure sensor with optimized size and doping concentration was fabricated with p-type (100) SOI wafer by using the standard CMOS lithography process and anisotropic wet-etch release process. The double-sided polished SOI wafer has a 200 nm buried oxide (BOX) layer and a 100 nm top silicon layer. Figure 8a–h briefly shows the fabrication process of this pressure sensor.

Firstly, Boron ions of 20 keV energy and 5 × 10^1^^3^ Dose/cm^2^ were implanted on the top silicon layer for the piezoresistance of the SiNW, followed by annealing for dopant activation. Then, the extra SiO_2_ layer of 50 nm and silicon nitride layer (SiN_x_) of 100 nm were deposited on both sides of SOI wafer by using low pressure chemical vapor deposition (LPCVD). The compressive stress formed in the SiO_2_ adhesive layer compensates the tensile stress in the SiN_x_ layer. For SiNW pattern and electrical isolation, they were patterned and the top silicon layer was etched by deep reactive ion etching (DRIE), and the SiO_2_ in the SOI wafer provides an etch stop barrier. In order to fabricate metal pad, after etching extra SiO_2_ and SiN_x_ layers on front side, a 1.5 µm thick Aluminum was deposited onto the silicon contact surfaces while the Al pad is patterned by using a liftoff process. Besides, SiNW structures like bridge shape were released through buffered oxide etcher (BOE) solution and critical point drying. In order to protect against corrosion of Aluminum structures, they are covered by thick photoresist mask. After that, the tetramethyl ammonium hydroxide (TMAH) wet etch is conducted to release the diaphragm structure. The substrate of the SOI wafer is ground to 675 µm, and backside of the substrate was gradually etched by 363 K TMAH in many etch steps to obtain diaphragm less than 6 µm thickness. Finally, the extra SiO_2_ and SiN_x_ mask layers on backside were removed using BOE solution and CF_4_ gas, and then a Pyrex glass 7740 wafer of 500 µm thickness was placed on the etched backside of the SOI wafer and anodically bonded by applying an electric field (900 V DC) across the bonding interface at 360 °C. The bonding process is carried out in a vacuum environment.

**Figure 9:**
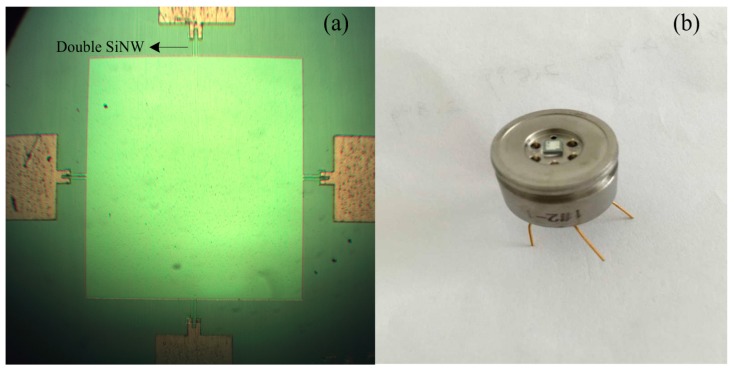
(**a**) A photograph of the fabricated SOI pressure sensor die using double SiNW before the release; and (**b**) a photograph of packaged pressure sensor.

Figure 9a displays the fabricated SOI piezoresistive pressure sensor die using double SiNW of 1400 nm × 100 nm cross area and 10 µm length. In addition, semi-hermetic diaphragm packaging structures are employed as shown in Figure 9b. In the following section, we will examine the performance of the pressure sensor.

## 5. Experimental Section and Discussion

### 5.1. Experimental Setup

**Figure 10:**
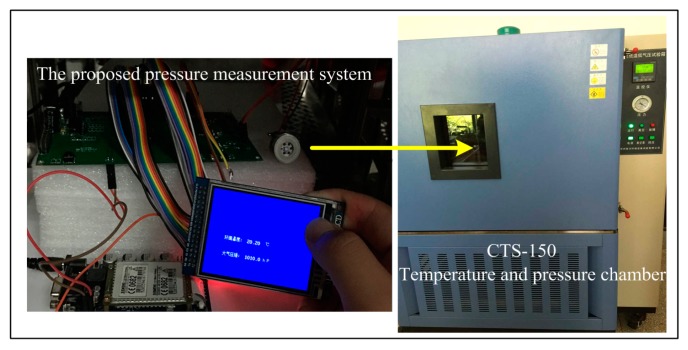
Photographs of the proposed SiNW pressure measurement system and calibration and test experimental setup. The CTS-150 constant temperature and pressure chamber is manufactured by Cliphyco Instruments Co., Limited (Hong Kong, China).

The test experiments are conducted to evaluate and optimize the performance of the sensor. Figure 10 shows the proposed SiNW pressure measurement system and the CTS-150 constant temperature and pressure chamber manufactured by Cliphyco Instruments Co., Limited (Hong Kong, China), which can well meet the calibration requirements of pressure sensor. Meanwhile, the effect of temperature on the pressure sensor can also be investigated by using this temperature-programmable chamber. The calibration and test procedures are as follows: Firstly, the SiNW pressure sensor without calibration is put into the CTS-150 constant temperature and pressure chamber, which is used to produce the standard calibration pressure that we want to obtain, such as 10, 20, 30, 40 kPa and so on. Applied pressure changes the resistance of SiNW. Depend on the changed resistance and fixed current, and then pressure voltage values and temperature voltage value are obtained by means of our SiNW pressure sensor and temperature sensor using voltage measurement. Each sample data (output voltage of the sensor) for each temperature and pressure are repeatedly measured three times continuously and averaged to reduce random errors. Secondly, the temperature in the experimental chamber is changed in the wide ranges in order to facilitate calibration pressure and temperature drift compensation. Thirdly, the data fusion algorithm based on the PSO–BP is introduced to establish the function relationship between the standard calibration pressures obtained by the chamber and the pressure voltage values as well as the temperature values obtained by our fabricated pressure sensor in a wide range of temperature and pressure. Furthermore, the PSO–BP algorithm and the function relationship, which minimizes the sensor errors due to the temperature drift, are written into the embedded system that allows digital pressure sensor real-time and online display pressure data in thin-film transistor (TFT) screen or send pressure data to the mobile terminal by the global system for mobile communication (GSM) module. Finally, we conduct the experiments to test the prediction performance of the PSO–BP algorithm based on the comparison between data that are not processed by algorithm and the data that are processed by the PSO–BP algorithm.

### 5.2. Sensor Output Result

**Figure 11:**
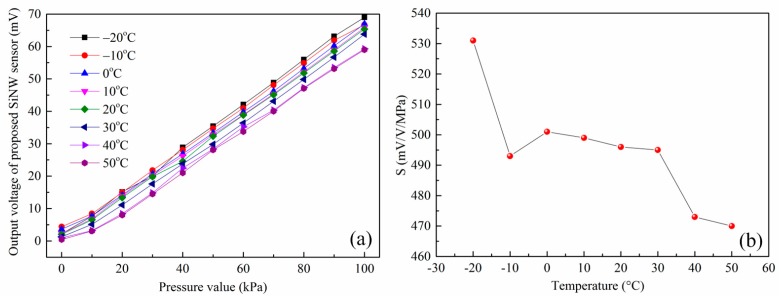
(**a**) Dependence of the output voltages of SOI pressure sensor without compensation on the pressure with temperature from −20 to 50 °C; and (**b**) the relationship curve between the sensitivity and temperature.

The sensitivity analysis is the study of change in output voltage with respect to the applied pressure. Figure 11a illustrates the relationship between the output voltage value of the sensor and the standard pressure under different temperatures. It is observed that the output voltage increases substantially linearly with the applied pressure. However, the output curves of the sensor at different temperatures are not coincident and there are also differences in the zero output voltage as well as the sensitivity, which demonstrates that designed SiNW pressure sensor has a temperature drift and is consistent with the conclusion of the literature [12,27,32]. The lower the temperature is, the greater the output voltage is. Therefore, the sensitivity of the piezoresistive SiNW sensor has a negative temperature coefficient. To be more intuitive, Figure 11b displays the sensors sensitivity change with temperature increasing. It is found that the sensitivity decreases substantially as the temperature increases and the TCS extracted is around −6.4 × 10^−4^ K^−1^ by linear fitting and is in the same order of magnitude as the theoretical predicted value. As discussed in the previous theoretical study, this is mainly due to the fact that the piezoresistive coefficient decreases with increasing temperature. For the constant-current case (0.05 mA, around 5 V equivalent voltage), sensor of the released double SiNW measured by a quarter Wheatstone bridge has sensitivity of 495 mV/V·MPa at room temperature and it has 1.4 times more sensitivity than the released single SiNW pressure sensor [7], which matches well with the previous theoretical predictions.

One notes that the sensitivity of the proposed SiNW piezoresistive pressure sensor in this paper can be increased to more than four times as compared to that of SiNW pizoresisitve pressure sensor without the giant piezoresistive effect [12], which can be attributed to the obvious enhanced surface effect and corresponding giant piezoresistive effect of SiNW [31,32,33,34]. Recently, Sun et al. measured the piezoresistive coefficients of a series of SiNWs with different length and width [40]. It is also confirmed that there are giant piezoresistive properties in the silicon nanofilms, and their piezoresistive coefficients can increase even up to 1203 × 10^−11^ Pa^−1^, which is much higher by one order of magnitude than that of bulk silicon. They believe that the residual stress in the silicon wires with different widths has a very critical impact on their piezoresistive coefficient. However, due to various constraints, we do not carry out a more detailed analysis of the impact of residual stress on sensitivity of the pressure sensor. In order to ensure the measuring precision of the piezoresistive pressure sensor, we have proposed a compensation method based on the PSO–BP neural network to solve the temperature drift of the sensor and achieve a linear output in the entire measuring range.

### 5.3. Data Fusion Using PSO–BP Algorithm

#### 5.3.1. PSO–BP Neural Network Algorithm Overview

The hybrid PSO–BP algorithm combining the global PSO algorithm with the BP neural network includes two aspects: one is using the PSO algorithms to optimize the BP neural network’s connection weights between each layer, and the other is using PSO algorithms to optimize the topology of BP neural network [28,29]. This paper mainly applied PSO algorithms for neural network training, which can avoid the disadvantage of easily trapped into local optimum of BP algorithm. The fundamental idea for the PSO–BP algorithm is to combine the fast global search ability of the PSO algorithm with the fast local search ability of BP neural network, so as to avoid getting into infinitesimal locally effectively and keep the merits of high prediction precision and rapid convergence. Distinctly, the PSO–BP algorithm is not discussed in detail, for it has detailed description in References [28,29].

**Figure 12:**
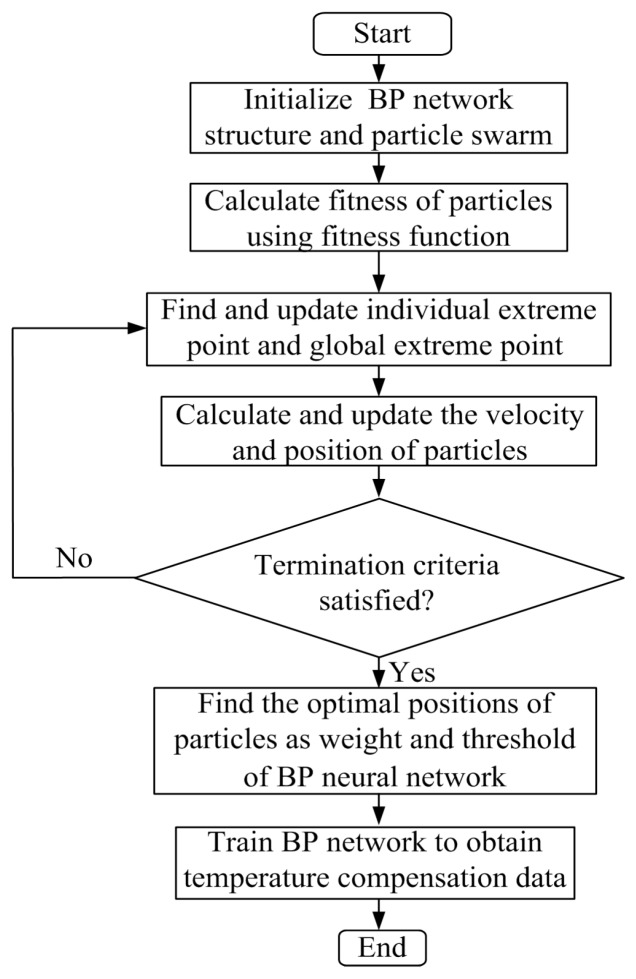
Flowchart depicting the particle swarm optimization–back-propagation (PSO–BP) neural network algorithm.

As illustrated in Figure 12, the main ideal of the PSO–BP compensation algorithm can be summarized as follows: In the first place, the related parameters of PSO–BP neural network are set, such as the number of layers of neural network, the number of neurons in each layer and dimension of particle. The positions and velocities of a group of particles are randomly initialized in the range of [0, 1]. Secondly, the PSO–BP is trained by using the initial particle’s position, and each initialized particle’s fitness value is evaluated by using the fitness function and the learning error between the BP network output and the desired output is also calculated. Thirdly, the fitness value of each individual particle is compared with individual extreme value (that is, fitness value of the individual extreme point), if the fitness function value is smaller, the individual extreme point and global extreme point of particles will be updated or selected. Fourthly, the positions and velocities of all the particles are updated, while a group of new particles are generated, and then each new particle’s fitness value is evaluated, meanwhile, the learning error is also recalculated. The learning error at current epoch will be reduced by changing the particles position, which will update the weight and threshold of the network. Fifthly, this process is repeated until the termination criteria either minimum learning error or maximum number of iteration is satisfied. Then, it can find the optimal positions of particles and best weight and threshold. Finally, BP neural network is trained to obtain temperature compensation data by using optimized weight and threshold. It is noteworthy that the training’s goal of BP neural network is to obtain a minimum sum of absolute error between the prediction and the sample desired output value. In this paper, the fitness function is described as the following formula:

(7) $$\mathrm{fitness}={\sum_{i=1}^{k}\left|p(i)-d(i)\right|}^{2}$$

where *k* refers to iterations of PSO–BP algorithm, *p*(*i*) is the network’s prediction output, and *d*(*i*) is the calibration value.

#### 5.3.2. Temperature Compensation by PSO–BP Data Fusion Algorithm

According to the calibration requirement of the pressure sensor, the calibration output voltages of the SOI pressure sensors under different pressures and temperatures (the calibration sample data) for training PSO–BP compensation algorithm are measured three times repeatedly, and the average values are listed in Table 1, which try to avoid incorporating the random errors into the temperature compensation using the PSO–BP data fusion method. The output standard pressure of the CTS-150 constant temperature and pressure chamber is marked as *P*, and the *U_p_* represents the average output voltage of our sensor. According to Table 1, it is found that the *U_p_* changes with temperature *T* and hence there is a drift in the sensor output characteristics. The temperature drift can especially cause a few mV errors at full scale. Obviously, the pressure sensor is seriously influenced by the temperature. Then, MATLAB (version R2013b, MathWorks, Natick, MA, USA) is applied to establish a data fusion model based on the PSO–BP algorithm and process these training sample data. The training data must be pre-processed to prevent nodes quickly reaching a saturated state and becoming unable to continue learning. According to the “mapminmax” function of MATLAB, the normalized formula is as follows:

(8) $$y=\frac{\left(y_{\max}-y_{\min})\times (x-x_{\min}\right)}{\left(x_{\max}-x_{\min}\right)}+y_{\min}$$

If the data are normalized to (−1, 1), *y*_max_ is 1 and *y*_min_ is −1. *x* is the output value of SOI pressure sensor at a reference temperature, while *x*_max_ and *x*_min_ are the maximum and the minimum output values, respectively. On the basis of this formula, the training data in Table 1 can be normalized. Theoretical studies have proven that the three-layer neural network can realize any complex nonlinear mapping problem, so the prediction model adopts a three-layer BP neural network with a single hidden layer for temperature compensation. This paper sets the input layer with two nodes (corresponding to the temperature signal and pressure signal without compensation), the hidden layer with five nodes, and the output layer with one node (corresponding to pressure signal after compensation). The maximum training times is 1500, the target error is 5 × 10^−6^, the training function is “trainlm”, the learning function is “learngdm” and the learning rate is set to 0.01. The population size is set to 40, the inertia weight is set to 1, and learning factor is chosen to be 2.5. After the optimization algorithm terminates, connection weights between the input layer and hidden layer ω_1_, connection weights between the output layer and hidden layer ω_2_, scale factor *b*_1_ and shift factor *b*_2_ is:

$$\omega_{1}=\left\{\begin{array}{c} 0.5609\\ -0.5605\\ -0.5400\\ 0.2037\\ -0.6102\\ -0.3668\\ 0.5410\\ 0.3031\\ 0.9639\\ 2.991 \end{array}\right\},\;\omega_{2}=\left\{\begin{array}{c} 0.0067\\ 1.2784\\ 0.7569\\ 1.1558\\ 0.06218 \end{array}\right\},\;b_{1}=\left\{\begin{array}{c} -0.4122\\ 2.0836\\ -2.1011\\ -0.0698\\ 1.3690 \end{array}\right\}\;\mathrm{and}\;b_{2}=\left\{-0.3886\right\}.$$

Once the connection weights and other important parameters are obtained, the PSO–BP data fusion algorithm can be ported to the 32-bit STMicroelectronics (STM32) microprocessor in the form of C language procedures. In this case, the proposed the SiNW pressure measuring system can measure and give the actual pressure with temperature compensation in real time.

**Figure 13:**
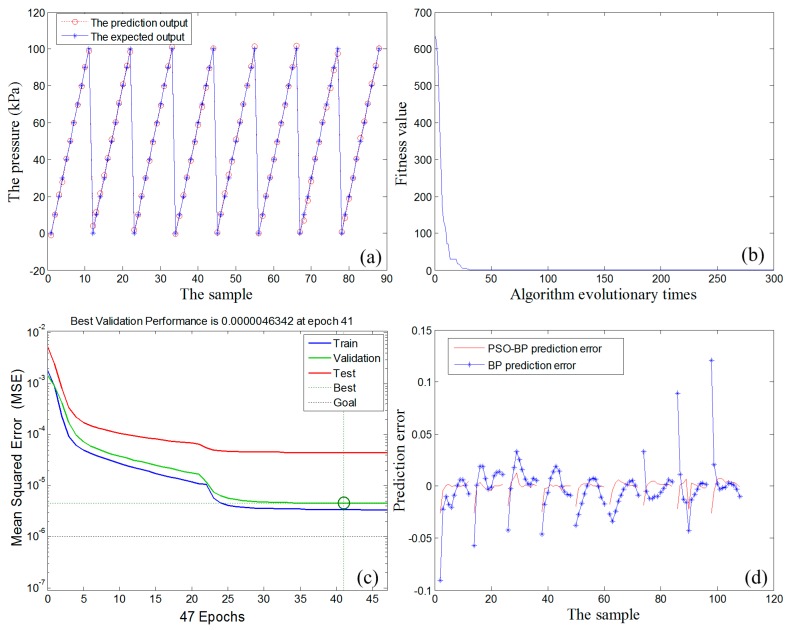
(**a**) The prediction output of the PSO–BP neural network algorithm; (**b**) fitness curve; (**c**) mean-squared error curve of the PSO–BP neural network algorithm; and (**d**) error curves of BP algorithm and the PSO–BP algorithm

Figure 13 summarizes the temperature compensation results of the proposed SiNW pressure sensor through the PSO–BP data fusion algorithm. Specifically, Figure 13a exhibits the relationship curves of the prediction output of PSO–BP neural network (circle) and the expected output (asterisk) coincide with each other, which indicates that the PSO–BP neural network can significantly weaken the influence of temperature and predict the pressure value of the sensor chip with the minimum error. In Figure 13b, it is found that the fitness of PSO algorithm has reached the minimum before 50 iterations. In other words, it has converged to the optimal weight and threshold. As plotted in Figure 13c, the target error reaches 4.6342 × 10^−6^ after 41 iterations of the training cycle. However, the calculation shows that the BP algorithm needs to be iterated 122 times to achieve similar target error, which manifests that PSO–BP algorithm significantly improves the operating efficiency. Figure 13d gives the temperature compensation errors of PSO–BP neural network and traditional BP neural network. It is clear that the compensation accuracy of PSO–BP neural network is significantly higher than that of the BP network.

**Figure 14:**
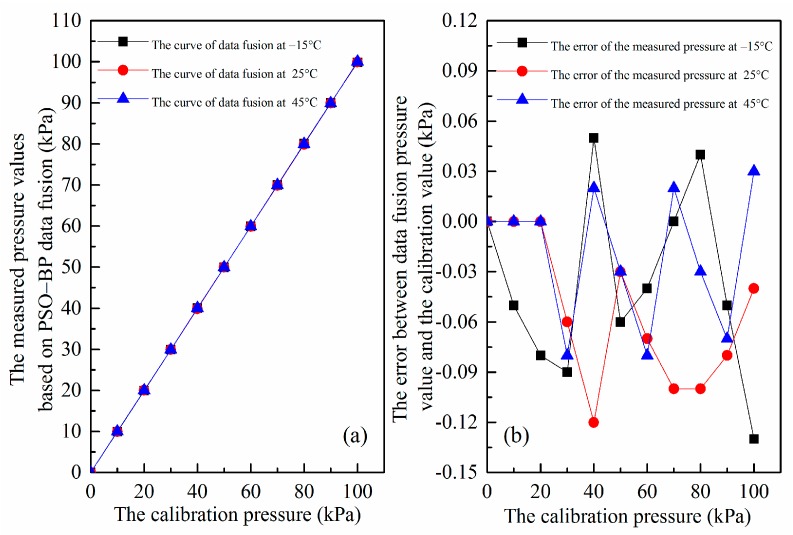
(**a**) The relationships between the actual measurement pressures of the digital SiNW pressure sensor designed in the paper and the calibration pressure at different temperature; and (**b**) the absolute error between the corrected pressures and the calibration pressures at different temperature.

However, we need to further verify this compensation algorithm at actual situation and to ensure reliability under the other temperatures (45, 25 and −15 °C are chosen) different from the samples. The results are plotted in Figure 14. Figure 14a illustrates the relationship curves between the corrected output pressures of our SiNW pressure sensor through the genetic algorithm-wavelet neural network (GA-WNN) data fusion and the calibration pressures at different temperatures, which coincide together and are highly linear over the 0–100 kPa range. This result indicates that the influence of the temperature can be neglected once the PSO–BP algorithm is applied. Figure 14b gives the absolute error between the output pressure that is compensated by the PSO–BP data fusion algorithm and standard calibration pressure obtained by CTS-150 constant temperature and pressure chamber. The maximal prediction error after compensation of the PSO–BP algorithm is only 0.13 kPa, which demonstrates that the PSO–BP algorithm can study and predict the internal rule between piezoresistive coefficient and temperature based on experimental sample data and this compensation method is accurate and effective to deal with temperature drift. Thus, the digital SiNW pressure sensor is able to achieve high measurement accuracy.

## 6. Conclusions and Future Work

In this paper, the SOI piezoresistive pressure sensor employing the high piezoresisitive effect of double SiNW is proposed and optimized by ANSYS finite element method. The use of the SiNW enables the designed pressure sensor to have the high sensitivity and reduced sensor size. Our studies firstly present a comprehensive analysis of sensor size optimization and doping concentration optimization according to the demands of high sensitivity. We also explore the relationship of SiNW size and doping concentration with noise voltage and SNR. Considering the stress simulation and safety limitations of fabrication process, the proposed high performance piezoresistive pressure sensor is designed to own the nanowire size under 1400 nm × 100 nm, the nanowire length of 10 µm, the membrane top-layer thickness of 100 nm, the middle silicon dioxide layer thickness of 200 nm, the diaphragm size of 200 µm × 200 µm, and the remaining silicon thickness of 6 µm. Then, manufacturing process is given and the optimized double SiNW pressure sensor is fabricated, and its sensitivity can be increased to more than four times as compared to that of SiNW pizoresisitve pressure sensor without the giant piezoresistive effect, which can be attributed to the obvious enhanced surface effect and corresponding giant piezoresistive effect of SiNW. However, the experimental results also show that the output signal of SOI piezoresistive pressure sensor and its zero point and sensitivity will drift with temperature change. Therefore, we offer a versatile compensation method based on data fusion by using the PSO–BP algorithm for compensating and correcting the outputs of the sensor. The results of comparative experiments confirm that the compensation method is effective and can greatly improve the measuring accuracy.

## Figures and Tables

**Table 1:** The calibration sample data of the SiNW pressure sensor for the training of the PSO–BP compensation algorithm, which are obtained with pressures from 0 to 100 kPa and with temperatures from −20 to 50 °C.

	Pressure	*P* = 0 kPa	*P* = 10 kPa	*P* = 20 kPa	*P* = 30 kPa	*P* = 40 kPa	*P* = 50 kPa	*P* = 60 kPa	*P* = 70 kPa	*P* = 80 kPa	*P* = 90 kPa	*P* = 100 kPa
Temperature												
***T* = −20 °C**		*U_p_ =* 2.20 mV	*U_p_ =* 7.68 mV	*U_p_ =* 15.12 mV	*U_p_ =* 20.00 mV	*U_p_ =* 28.88 mV	*U_p_ =* 35.44 mV	*U_p_ =* 42.11 mV	*U_p_ =* 48.88 mV	*U_p_ =* 56.00 mV	*U_p_ =* 63.13 mV	*U_p_ =* 68.96 mV
***T* = −10 °C**		*U_p_ =* 4.40 mV	*U_p_ =* 8.44 mV	*U_p_ =* 14.96 mV	*U_p_ =* 21.81 mV	*U_p_ =* 28.12 mV	*U_p_ =* 34.72 mV	*U_p_ =* 41.00 mV	*U_p_ =* 48.08 mV	*U_p_ =* 54.89 mV	*U_p_ =* 61.88 mV	*U_p_ =* 66.71 mV
***T* = 0 °C**		*U_p_ =* 3.64 mV	*U_p_ =* 7.73 mV	*U_p_ =* 14.16 mV	*U_p_ =* 20.71 mV	*U_p_ =* 26.89 mV	*U_p_ =* 33.35 mV	*U_p_ =* 39.80 mV	*U_p_ =* 46.20 mV	*U_p_ =* 53.20 mV	*U_p_ =* 60.22 mV	*U_p_ =* 66.96 mV
***T* = 10 °C**		*U_p_ =* 2.29 mV	*U_p_ =* 6.84 mV	*U_p_ =* 13.85 mV	*U_p_ =* 20.19 mV	*U_p_ =* 26.16 mV	*U_p_ =* 32.80 mV	*U_p_ =* 38.96 mV	*U_p_ =* 45.39 mV	*U_p_ =* 52.21 mV	*U_p_ =* 58.95 mV	*U_p_ =* 65.81 mV
***T* = 20 °C**		*U_p_ =* 2.04 mV	*U_p_ =* 6.56 mV	*U_p_ =* 13.36 mV	*U_p_ =* 19.84 mV	*U_p_ =* 24.56 mV	*U_p_ =* 32.33 mV	*U_p_ =* 38.84 mV	*U_p_ =* 45.12 mV	*U_p_ =* 51.82 mV	*U_p_ =* 58.60 mV	*U_p_ =* 65.36 mV
***T* = 30 °C**		*U_p_ =* 1.28 mV	*U_p_ =* 5.11 mV	*U_p_ =* 11.11 mV	*U_p_ =* 17.60 mV	*U_p_ =* 23.80 mV	*U_p_ =* 29.84 mV	*U_p_ =* 36.40 mV	*U_p_ =* 43.11 mV	*U_p_ =* 49.84 mV	*U_p_ =* 56.68 mV	*U_p_ =* 63.80 mV
***T* = 40 °C**		*U_p_ =* 0.97 mV	*U_p_ =* 3.20 mV	*U_p_ =* 8.40 mV	*U_p_ =* 14.84 mV	*U_p_ =* 22.47 mV	*U_p_ =* 28.36 mV	*U_p_ =* 35.20 mV	*U_p_ =* 40.38 mV	*U_p_ =* 47.29 mV	*U_p_ =* 53.48 mV	*U_p_ =* 59.29 mV
***T* = 50 °C**		*U_p_ =* 0.44 mV	*U_p_ =* 3.08 mV	*U_p_ =* 8.00 mV	*U_p_ =* 14.49 mV	*U_p_ =* 21.00 mV	*U_p_ =* 28.12 mV	*U_p_ =* 33.75 mV	*U_p_ =* 40.04 mV	*U_p_ =* 47.09 mV	*U_p_ =* 53.15 mV	*U_p_ =* 59.00 mV
